# Advances in Molecular Mechanisms and Immunotherapy Involving the Immune Cell-Promoted Epithelial-to-Mesenchymal Transition in Lung Cancer

**DOI:** 10.1155/2019/7475364

**Published:** 2019-08-19

**Authors:** Serena De Matteis, Matteo Canale, Alberto Verlicchi, Giuseppe Bronte, Angelo Delmonte, Lucio Crinò, Giovanni Martinelli, Paola Ulivi

**Affiliations:** ^1^Biosciences Laboratory, Istituto Scientifico Romagnolo per lo Studio e la Cura dei Tumori (IRST) IRCCS, Meldola, Italy; ^2^Department of Medical Oncology, Istituto Scientifico Romagnolo per lo Studio e la Cura dei Tumori (IRST) IRCCS, Meldola, Italy; ^3^Scientific Directorate, Istituto Scientifico Romagnolo per lo Studio e la Cura dei Tumori (IRST) IRCCS, Meldola, Italy

## Abstract

Immunotherapy has offered a new opportunity for the treatment of many malignancies. In patients with lung cancer, immune checkpoint inhibitors have significantly improved survival. However, little is known about predictive factors or primary and acquired resistance mechanisms. Epithelial-to-mesenchymal transition (EMT) is a complex of phenotypic changes involved in carcinogenesis and resistance to cancer treatments. Specifically, immune cells in the tumor microenvironment can promote EMT, and mesenchymal phenotype acquisition negatively regulates the anticancer immune response. EMT is associated with higher expression of PD-L1 and other immune checkpoints. In this review, we focused on the role of EMT in the interplay between tumor cells and the immune system, with particular emphasis on lung cancer. On the basis of our findings, we hypothesize that the effects of EMT on immune cells could be overcome in this disease by a new combination of immune checkpoint inhibitors.

## 1. Introduction

The epithelial-mesenchymal transition (EMT) plays a key role in the transdifferentiation process during solid cancer development. The acquisition of a migratory mesenchymal phenotype by tumor cells primarily involves several signaling cascades, including transforming growth factor-*β* (TGF-*β*), cadherin, Notch, and WNT/*β*-catenin pathways [1]. Recently, reports investigated how the acquisition of mesenchymal features by tumor cells could also lead to the development of an inflammatory or immunosuppressive microenvironment [2]. Moreover, there is evidence that EMT plasticity is supported by the dynamic crosstalk between cancer cells and immune cells, favoring tumor growth, angiogenesis, and metastasis. The link between cancer and inflammation involves a series of signaling networks between different cellular components of the tumor site, via the activity of soluble immune-mediators [2].

In the light of current knowledge, cancer cells undergoing EMT may affect the cellular components within the tumor microenvironment (TME), including immune cells.

The present paper reviews the role of EMT in the interplay between tumor cells and the immune system, highlighting the molecular mechanisms that promote tumor development and aggressiveness and focusing in depth on putative strategies to overcome EMT-mediated resistance to epidermal growth factor receptor (EGFR) and tyrosine kinase inhibitors (TKIs) in lung cancer. Immunotherapy has become a standard part of therapy for patients with advanced lung cancers, with several anti-PD1 and/or anti-PD-L1 drugs for non-small cell lung cancer (NSCLC). EMT that induces checkpoint-dependent resistance to anti-tumor immunity may render cancer cells nonresponsive to therapies targeting one or few checkpoints (e.g., anti-PD-L1 and anti-CTLA-4) [3]. Here, we also report recent literature findings on the mechanisms through which EMT may influence antitumor immune response and, consequently, the efficacy of immunotherapy in lung cancer.

### 1.1. Epithelial Mesenchymal Plasticity Regulates the Function of Different Immune Cells in Organ-Specific Metastasis

Organotropism metastasis mechanism is one of the most unanswered questions in the field of cancer research and is regulated by multifaceted factors including intrinsic properties of cancer cells, features of organ microenvironments, and cancer cell-organ interactions. EMT is recognized as an initial and critical event for the metastasis of carcinomas. Clinical achievements of cancer immunotherapy currently overcome our scientific understanding of the immune-related mechanisms for organotropic metastasis.

Mechanisms underlying the regulation of the sensitivity of organ-specific metastasis versus primary tumors to immunomodulation are understudied. However, the heterogeneity of tumor immune landscapes both locally and systemically could be partly attributed to the tumor epithelial-mesenchymal plasticity in modulating antitumor immunity from tumor microenvironment components [4].

It has been reported that triple-negative breast cancer cell-derived exosomes induced the polarization of macrophages towards an M2-phenotype, creating the conditions for lymph node metastasis [5].

A recent study by Chockley et al. reported that NK cells were activated to attack metastatic EMT tumor cells through the balance of activating and inhibitory receptors engaged by different ligands, and the EMT-induced NK cell activity mediated immunosurveillance in lung metastasis [6].

It turns out that epithelial-mesenchymal plasticity is an essential factor in governing metastasis and regulating the function of different immune cells in organ-specific metastasis.

## 2. Role of the Immune System in Lung Cancer

It is known that chemotherapy and radiotherapy induce a durable anticancer immune response. This unusual phenomenon occurs via a modification of tumor immune infiltrate, with a consequent shift from a preexisting immune response to a therapy-induced immune response. The recent availability of immune checkpoint inhibitors has enabled us to directly modify immune response through the impairment of immune tolerance towards tumor antigens [7]. As some studies on carcinogen-initiated sarcomas have demonstrated in mice, high tumor mutational burden induced by carcinogens favors the development of neoantigens, providing a good substrate for T-cell priming and leading to a cytotoxic antigen-specific elimination [8]. The hypothesis that an impaired immune system may play a role in cancer initiation and progression is supported by evidence that cancer incidence is higher in patients suffering from chronic inflammatory conditions, in immune-compromised individuals and in those with age-related immune senescence [9, 10].

Some patients with NSCLC also suffer from chronic obstructive pulmonary disease (COPD) characterized by an inflammation-rich microenvironment (mainly “neutrophilic” inflammation) that may support carcinogenesis [11]. Immune cells represent 50%–75% of the cellular content of tumor specimens in these patients, almost threefold the number of immune cells identified in nonadjacent lung tissue [12]. In addition to cytotoxic T cells, the immune infiltrate includes numerous cellular components that have a varying effect on immune response and, consequently, on prognosis.

Neutrophils are the most abundant immune cell type in NSCLC, accounting for around 20% of the immune cell infiltrate, while the percentage of macrophages is lower in lung cancer specimens compared to noncancerous lung tissue [13]. Neutrophils play an important role in tumor initiation, releasing oxygen- and nitrogen-free radicals that promote DNA point mutations and genetic instability [14]. A proinflammatory phenotype has been described in early-stage lung cancer and associated with a high production of several chemokines. These tumor-associated neutrophils appear to have an anti-tumor effect by stimulating T-cell proliferation and interferon- *γ* (IFN-*γ*) release [15]. Conversely, it has also been seen that a neutrophil-rich infiltrate negatively correlates with tumor-infiltrating CD8^+^ and CD4^+^ cells, efficiently predicting patient mortality in NSCLC [13].

Another 10% of the immune infiltrate in NSCLC specimens display a cellular phenotype characterized by the positive CD45 and CD33 expression and negative CD14, CD68, and CD66b expression. These cells may represent an “early” myeloid-derived suppressive cell (MDSC) subset [16, 17]. MDSCs have emerged as potentially therapeutically important immune-suppressive cells within EMT [18].

Regulatory T cells (Tregs) are a heterogeneous population of T lymphocytes, identifiable as CD4^+^ CD25^+^ FOXP3^+^ T cells, which contribute to immune tolerance. A high percentage of tumor-infiltrating Tregs is associated with poor prognosis in NSCLC patients [19]. Tertiary lymphoid structures (TLS), characterized by a T-cell zone with lysosome-associated membrane glycoprotein 3 (LAMP) positive mature dendritic cells (DC^–^LAMP^+^) and a follicular zone in which B lymphocytes actively proliferate and differentiate in germinal centers, are found in around 70% of NSCLC patients, and their high density correlates with favorable prognosis [20].

Natural killer (NK) cells show an intrinsic cytotoxic activity against cancer cells, but the function of these cells may be compromised by cigarette smoking, with a consequent impact on lung carcinogenesis [21]. A positive correlation between NK count and outcome has been observed in both early and advanced NSCLC patients [22, 23]. Moreover, NK cytotoxic function appears to be augmented by their interaction with alveolar macrophages, which constitute the most numerous component of the tumor microenvironment. A high density of M1 macrophages correlates with a favorable prognosis in NSCLC [19].

Three classes of tumor immune microenvironment (TIME) have been identified with regard to the composition of immune cell infiltration: (a) infiltrate-excluded EMT (I-E), which is rich in immune cells but void of cytotoxic lymphocytes (CTL) in the core; (b) infiltrated-inflamed (I-I), with a high infiltration of CTLs; (c) infiltrated-TLS, a subclass of the infiltrated-inflamed microenvironment which show an infiltration of B cells, dendritic cells, and Tregs [24]. EMT has a dynamic evolution in relation to tumor genotype and phenotype because some oncogenes promote an immunosuppressive microenvironment that facilitates tumor development and growth [25, 26]. Thus, the tumor immune infiltrate is very heterogeneous, and these results in different types of tumor immune microenvironment could influence the onset of EMT of cancer cells.

In the following sections, we review the role of genomic and phenotypic changes in immune response regulation within the context of EMT.

## 3. Dynamic Crosstalk between Cancer Cells and Immune Microenvironment Supports EMT Plasticity

### 3.1. EMT Regulation via Tumor-Infiltrating Immune Cells

Inflammation plays a crucial role in each stage of tumor development. Cancer cells evade immune surveillance, altering the balance between cytokine- and chemokine-mediated proinflammatory and anti-inflammatory processes that modulate the recruitment, expansion, and function of immune cells constituting tumor-associated and systemic inflammation. The link between cancer and inflammation involves a series of signaling networks between different cellular components of the tumor site, via the activity of soluble immune mediators. These inflammatory mediators including interleukin-6 (IL-6), the chemokine IL-8, and tumor necrosis factor (TNF-*α*) promote the recruitment of immune cell populations to the tumor site [2]. One of the consequences of tumor-driven inflammation is the EMT induction, which endows the tumor cells with acquired mesenchymal phenotype of high motility, invasiveness, and ability to disseminate and metastasize [2].

**Figure 1:**
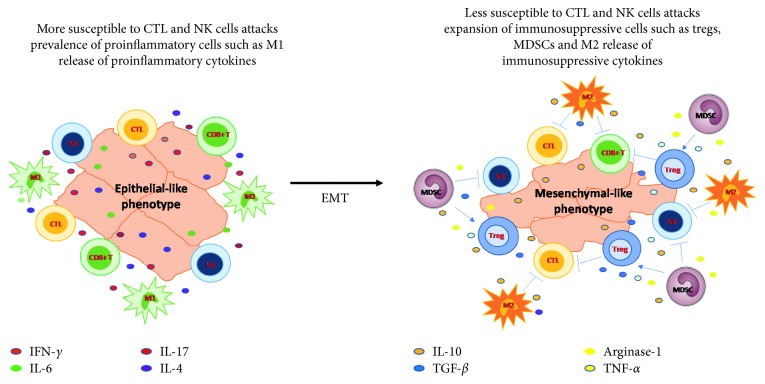
Mesenchymal phenotype is characterized by a loss of susceptibility to cytotoxic T cells (CTL) and natural killer (NK) cells; the switch of tumor-associated macrophages (TAM) from M1-like proinflammatory to M2-like anti-inflammatory phenotype; the expansion of immunosuppressive cells such as myeloid-derived suppressive cells (MDSC), regulatory T cells (Tregs), and M2-TAM and the release of immunosuppressive cytokines such as TGF-*β*, TNF-*α*, IL-10, and arginase-1.

Mechanisms used by tumor cells undergoing EMT to interact with immune cells in promoting tumor growth, angiogenesis, and metastasis have been explored in several cancer models [27–31]. Immune contexture analysis of tumor tissue has shown that the acquisition of a mesenchymal phenotype is affected by the presence of a mosaic of immunosuppressive cells such as MDSCs, tumor-associated macrophages (TAMs) with M2-like phenotype, and Tregs [32–35]. Immunoregulatory enzymes and immunosuppressive cytokines released by these cells, such as IL-10, TGF-*β*, and TNF-*α*, inhibit NK cells, CD8^+^ T cells, and CTLs (Figure 1). The mesenchymal-like cancer cells can directly suppress the function of cancer-killing immune cells as well as promote the immunosuppressive microenvironment by recruiting or polarizing immune cells with immunosuppressive phenotype. For the interaction between mesenchymal-like cancer cells and immune cells, TGF-*β* signaling has been studied extensively. TGF-*β*, a well-known EMT inducer, can impair maturation, differentiation, and/or activation of both innate and adaptive immune cells. In several studies, it has been demonstrated that TGF-*β* secreted by cancer cells was able to polarize macrophages into M2-like ones, leading to suppression of the function of cytotoxic immune cells [36, 37]. In addition, TGF-*β* could downregulate the MHC class I proteins as reported in prostate cancer [38] as well as NSCLC cell lines [39].

EMT plasticity represents a unique feature exhibited by cancer cells in their adaptation to the changing microenvironment, due, in part, to a dynamic crosstalk with immune cells [40]. TAMs, one of the major components of the immune cell infiltrate, are involved in numerous tumor-promoting functions including EMT and immune suppression. In recent years, the role of TAMs in promoting the EMT has been investigated. Recently, Han et al. described some mechanisms by which TAMs are capable of promoting EMT, tumor cell growth, and migration in osteosarcoma cells. The authors demonstrated that TAMs may promote EMT *in vitro*, increasing the migratory capacity of osteosarcoma cells by STAT pathway activation or via the induction of COX-2, a crucial molecule in the inflammatory context [27].

The key role of TAMs in facilitating EMT progression has been also demonstrated in colorectal cancer cell lines via TGF-*β* secretion that leads to E-cadherin loss and high vimentin expression levels [41]. Furthermore, in another *in vitro* study, TAMs have been shown to enhance the aggressiveness of breast cancer HCC1954 cells via the IL-1*β*-dependent upregulation of COX-2 [42]. Li et al. reported that COX-2 regulates the interaction between MDSCs and nasopharyngeal cancer cells by triggering EMT on cell-to-cell contact, hypothesizing a novel strategy for suppressing metastases from nasopharyngeal cancer via COX-2 or MDSCs inhibition [43].

MDSCs are known to promote tumor invasiveness by supporting EMT [44]. Sangaletti et al. described one of the levels of tumor EMT regulation based on the interplay between MDSCs and EMT, highlighting the pivotal role of secreted protein that is acidic and rich in cysteine (SPARC), a master regulator of stromal remodeling, in promoting MDSCs expansion and recruitment needed for EMT in breast cancer cells. To better elucidate the role of MDSCs in EMT, the authors used a drug capable of interfering with their expansion and differentiation that led to a reversal of EMT in breast cancer mouse models [28].

Recently, Snail, a key transcriptional repressor of E-cadherin during EMT, was investigated for its role in promoting MDSC trafficking and tumor infiltration, with consequent suppression of antitumor immunity via CXCR2 in ovarian cancer cell lines [30]. Interestingly, some reports documented that Snail regulates other immune cells with immunosuppressive activity, including Tregs or TAMs [45, 46]. Platelets may also play an important role in EMT. In colon and breast cancer, platelets are known to induce EMT by promoting extravasation of cancer cells inducing EMT through direct contact and release of TGF-*β* [47]. In melanoma mouse models, these enucleated structures have been shown to increase the permeability of endothelial cells, promoting cancer cell extravasation [48].

There is still no evidence that T and B cells directly modulate tumor cell phenotype inducing EMT. Conversely, cancer cells undergoing EMT have been seen to activate immunosuppressive Tregs. A recent study, performed on gastric cancer (GC) cell lines and on patients, highlighted a new mechanism through which IL-15, secreted by GC mesenchymal stem cells in the microenvironment, upregulated the Tregs ratio and enhanced programmed cell death protein 1 (PD-1) expression in CD4^+^ T cells, promoting EMT [49].

The expression of Tregs and their correlation with the expression and transformation of epithelial-mesenchymal markers has been investigated in hepatocarcinoma (HCC) tissue. Shi et al. demonstrated that increased Tregs expression was associated with tumor metastasis and poor prognosis in HCC samples. The authors also observed a positive correlation between EMT markers (including vimentin, E-cadherin, and Snail) expressed by HCC cells and Tregs [50]. A study on cholangiocarcinoma revealed the involvement of Tregs in EMT induced by atypical protein kinase C-iota (aPKC-*ι*) through Snail regulation. Moreover, they reported that the overexpression of aPKC-I, its phosphorylated form, and Snail, as well as infiltrated Tregs, significantly correlated with poor prognosis [51].

In light of this, new hope is emerging that targeting immune components could change the natural history of these malignancies, through a better understanding of the molecular mechanisms by which EMT-inducing factors that may recruit specific subtypes of immune cells guide their differentiation and activation.

### 3.2. EMT Regulation by Tumor-Infiltrating Immune Cells in Lung Cancer

EMT has been associated with disease progression, poor prognosis, and immune evasion in lung adenocarcinoma (ADC) [52–54]. However, the extent to which EMT reprograms the tumor immune microenvironment is still largely unknown. Lou et al. reported an EMT-related mRNA signature associated with increased expression of immune inhibitory ligands and receptors in lung cancer [27]. Specifically, the authors found that EMT is highly associated with an inflammatory tumor microenvironment, increased expression of multiple immune checkpoint molecules (e.g., PD-L1, PD-L2 PD-1, T-cell immunoglobulin and mucin domain 3 (TIM-3), B- and T-lymphocyte attenuator (BTLA), and cytotoxic T-lymphocyte antigen 4 (CTLA-4)), and a high number of infiltrating Tregs. These data indicate that EMT may accelerate cancer growth and metastasis, not only by modulating cancer cells but also by reprogramming the immune response in the tumor microenvironment, suggesting its potential as a target for modulating response to immune checkpoint inhibitors. In addition, tumors harboring the EMT-signature displayed higher levels of Th1-inflammation markers than epithelial-like malignancies (e.g., IFN-*γ* and CXCL-10). Notably, tumors with both epithelial and mesenchymal features showed a comparable tumor mutational burden.

Moreover, TAMs also promote EMT of tumor cells by producing TGF-*β*, and analysis of 491 NSCLC patients revealed a positive correlation between intratumoral macrophage densities, EMT markers, intraepithelial TGF-*β* levels, and tumor grade [55]. A recent study have demonstrated that both ADC and squamous cell lung carcinoma with a mesenchymal phenotype are characterized by a TME in which an inhibition of antitumor T-cell functions is observed, with an upregulation of inflammatory cytokines and immunosuppressive immune checkpoint factors. These data evidenced that this phenomena occurs in lung cancer independently from the tumor histotype [56].

Although some studies associate PD-L1 overexpression with a higher response to immune checkpoint inhibitors, not all patients with high PD-L1 expression benefit from therapy, suggesting that microenvironment may influence response. PD-L1 expression is likely to be the consequence rather than the cause of increased tumor infiltration by immune cells [57], and it has been demonstrated that the transcription factor ZEB1, known to be an EMT driver, induces an upregulation of PD-L1 expression in tumor cells and enhances tumor response to IFN-*γ* [58]. A strong association between an active immune response and an increased number of immune checkpoint molecules in the tumor microenvironment has been observed in lung cancer patients undergoing EMT, indicating EMT as an independent mediator driving inflammation and immunosuppression. In line with the above observations, Gu et al. also showed that the infiltration of immune cells with antitumor function decreased in mesenchymal NSCLC, whereas that of immune cells with immunosuppressive function increased. In particular, the authors reported a lower infiltration of activated CD4^+^ T cells, effector CD4^+^ T cells, and Th17 cells, but a significantly higher infiltration of activated B cell and gamma delta T cells (*γδ*T). They also hypothesized that *γδ*T enhanced cell growth through IL-17 production and that IL-17 was involved in promoting EMT [59]. Several studies revealed an involvement of both innate and adaptive immune cells as drivers of EMT [60]. Recently, a study performed in mouse models, in contrast to the notion that EMT is associated with only tumor-promoting functions, demonstrated that EMT renders cancer cells more susceptible to NK cell cytotoxicity, contributing to the inefficiency of the metastatic process and opening up new possibilities for preventing metastasis by boosting NK cell functions [6]. aPKC has also been shown to induce the EMT process in NSCLC by interacting with TGF-*β* receptors, increasing Par6 phosphorylation, and thus regulating phospho-Par6-dependent EMT and cell migration [61].

These evidences suggest that EMT, triggered by immune cell subpopulations and several molecular modulators, could be able to modify the microenvironment of lung cancer, even though the molecular pathways still need to be elucidated.

## 4. EMT in Lung Carcinogenesis

EMT observed during embryogenesis (type I EMT) differs from EMT in fibrosis (type II EMT) and premalignant and malignant stroma (type III EMT) [62, 63]. In addition to lung cancer and chronic obstructive pulmonary disease (COPD) sharing biological phenomena including chronic inflammation, abnormal wound repair, extracellular matrix (ECM) degradation, angiogenesis, cell proliferation, and impaired apoptosis [64, 65], they are also both associated with the EMT process. COPD in current smokers is characterized by epithelial cells with a strong EGFR expression because of their activated state. Numerous slits are found in the reticular basement membrane (RBM) and cells residing therein express typical EMT markers, including matrix-metalloproteinase 9 (MMP9), vimentin, cytokeratin, and S100A4. Angiogenesis is also activated, RBM thus appearing highly vascularized as in type III EMT [66–68].

In subjects with COPD, the small airways undergo fibrosis and obliteration following type II EMT, a process mediated by fibroblasts and myofibroblasts, respectively. These mesenchymal cells are transformed through EMT. Consequently, in the ECM of these small airways, stiffness is impaired with obstruction during expiration [69]. Type III EMT appears to link lung cancer to COPD. In this specific process, proneoplastic stroma mediates the transformation of epithelial cells with smoke-related genetic mutations into cancer cells. Subsequently, both processes are exploited by epithelial cancer cells to locally invade and metastasize [70]. The use of corticosteroids by inhalation in COPD patients inhibits EMT in the large airways [71].

In lung ADC, sorafenib has been shown to suppress TGF-*β*-induced EMT through an increase in histone acetyltransferase and a decrease in histone deacetylase [72].

Our knowledge of these mechanisms supports the hypothesis that EMT is the key process in lung cancer development in COPD patients exposed to tobacco smoke.

## 5. Role of EMT in the Resistance of Lung Cancer Cells to EGFR-TKIs

The role played by EMT in the resistance to lung cancer therapy has already been studied for EGFR-TKIs. Sequist et al. reported the onset of EMT during treatment with EGFR-TKIs in some patients with no resistance mutations. Upon the development of resistance, a number submitted to rebiopsy showed EMT-related phenotypic changes. Although EMT was not present in patients with T790M EGFR mutation as a resistance mechanism, the original EGFR-activating mutation was retained [73]. Some preclinical studies have shed light on the intrinsic role of EMT in the resistance of cancer cells to EGFR-TKIs [74–78]. IGF-1R pathway activation impairs EGFR-TKI sensitivity through EMT as induction of the receptor leads to the acquisition of a mesenchymal phenotype [79]. EMT also mediates resistance to EGFR-TKIs by cigarette smoking through Src phosphorylation and is reversed by *N*-acetylcysteine [80, 81]. Soucheray et al. observed that EMT is mediated by the TGF-*β* pathway within the context of resistance to EGFR-TKIs. Mesenchymal clones show abundant secretion of TGF-*β*1, whereas MET-amplified cells produce less TGF-*β*1. When TGF-*β*1 is removed, an epithelial phenotype is partially restored and these cells recover sensitivity to EGFR-TKIs. TGF-*β*1 exposure is fundamental to maintaining the EGFR-TKI-resistant mesenchymal phenotype. TGF-*β*1 secretion increases in EGFR-mutant cells treated with an EGFR-TKI, but this effect is not observed in EGFR wild-type cells. When both EGFR and TGF-*β* receptor are inhibited, EMT is prevented but EGFR-TKI resistance develops through MET amplification or, more frequently, EGFR-T790M mutation. T790M mutation and EGFR activating mutation usually preexists in *cis* in a small population of cells [78, 82–84]. Some issues have yet to be clarified, e.g., the effect of EGFR inhibition on TGF-*β* secretion in EGFR-mutant cells and the reason why the mesenchymal phenotype and EGFR-TKI resistance are irreversible after TGF-*β*R inhibition. EGFR-TKI-resistant subpopulations with mesenchymal phenotype may preexist, and irreversible changes may be induced by prolonged EGFR-TKI treatment.

Yochun et al. recently used an *in vitro* model to study the role of the EMT-related transcription factor TWIST1 in the resistance to EGFR-TKIs. TWIST1 mediates resistance through the inhibition of apoptosis via the suppression of BIM expression. The genetic and pharmacological inhibition of TWIST1 helps to overcome both primary and acquired resistance to EGFR-TKI treatment [85]. However, the link between EMT and immune response regulation in oncogene-addicted lung cancers has yet to be clarified.

These data highlight the role of EMT-related phenotype in resistance to EGFR-TKIs in an alternative manner with respect to onset of T790M mutation, and it is a new emerging issue in the field of resistance to targeted therapy in NSCLC treatment.

## 6. EMT, Immune Reprogramming, and Immune Checkpoints

An interesting aspect is related to the mechanisms by which EMT can regulate PD-L1. Chen et al. identified a molecular link between EMT and more abundant expression of PD-L1 in human lung tumors, reporting that miR-200 downregulation and ZEB1 overexpression not only drives EMT but may also lead to PD-L1 upregulation [58]. In this study, the miR-200/ZEB1 axis has PD-L1 as a downstream target with consequent immunosuppression in the primary tumor. When PD-L1 is inhibited, immune infiltration is improved and tumor burden and metastases are reduced in mesenchymal tumors but not in epithelial tumors. Moreover, PD-L1 is synergistically regulated by IFN-*γ* stimulation and miR-200 repression [58].

Interestingly, high EMT marker expression has also been associated with high expression of immune checkpoints (including PD-1, PD-L1, and PD-L2), costimulatory receptors including OX40 (CD134), and its binding partner OX40-ligand (OX40L), CD137, TIM-3, lymphocyte activation gene 3 (LAG-3), and CTLA-4 [86]. Lou et al. confirmed these observations in 3 independent datasets of early and advanced NSCLC. Moreover, the authors found that tumors harboring high mesenchymal content were associated with increased infiltration by TILs and Tregs and increased expression of costimulatory molecules (such as CD80 and CD86). Tièche et al. observed a higher expression of PD-L1 and PD-L2 in mesenchymal paraclone cells of lung cancer cell line A549 with respect to epithelial and stem-like holoclone cells and phenotypically intermediate meroclone cells [87]. The relationship between PD-L1 expression and EMT has been found to be more evident in NSCLC patients with EGFR mutation than in those with wild-type EGFR. Moreover, EGFR-mutated NSCLC patients with a mesenchymal phenotype show higher levels of CD8^+^ and PD-1^+^ TILs. These findings suggest that PD-L1 overexpression in such patients may be a consequence of increased immune cell infiltration [88].

There is experimental evidence that the expression of a mesenchymal phenotype of cancer cells is associated with PD-L1 expression in several cancers [27] and with immune-resistance via multiple pathways [89]. This suggests that EMT plays a crucial role in immune-resistance and is a potent driver for the activation of an immunosuppressive network within the TME, including lung cancer.

**Figure 2:**
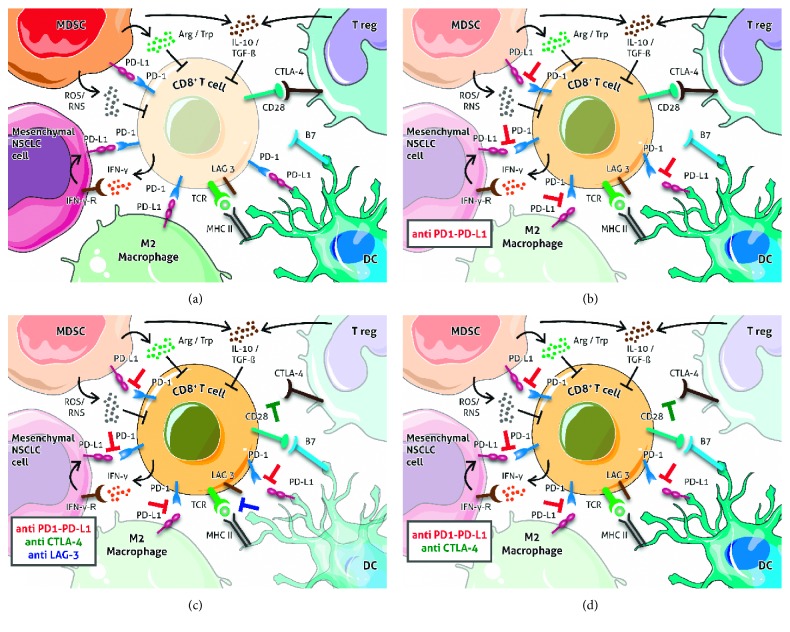
Hypothesis of the mechanism of action of immune checkpoint inhibitors (ICIs) combination. (a) Within the TME, CD8^+^ T cells antitumoral activity is inhibited through several molecular pathways. (b) Addiction of anti-PD-1/PD-L1 agents helps CD8^+^ T-cell reactivation, by blocking the PD-1/PD-L1 axis. (c) Addition of anti-CTLA-4 agents also inhibits Tregs, thus leading to the stimulatory binding of CD28 on CD8^+^ T cells with B7 on dendritic cells. (d) Further addition of anti-LAG3 agents could ultimately restore the CD8^+^ T cells activity against cancer cells, by enhancing T-cell receptor activity.

Another important topic linked to EMT in promoting immune escape relates to the expression of human leukocyte antigen class I (HLA-I). It has been reported that Snail is key to the downregulation of TGF -*β* - and epidermal growth factor- (EGF-) induced HLA-I in prostate cancer cells, leading to the attenuation of T cell-mediated lysis [38]. In this study, it has been reported that Snail knockdown does not fully reverse TGF-*β*-induced HLA-I downregulation. This suggests that other factors are involved in this process, namely, NF-*κ*B, which is the main transcription factor to activate HLA-I transcription. To some extent, TGF-*β* induces Snail binding with NF-*κ*B, which consequently cannot activate HLA-I and, finally, HLA-I is downregulated because of TGF- *β* activation [38]. Moreover, in breast cancer *in vitro* models, it has been demonstrated that mammary tumor cells arising from cell lines with a higher number of epithelial features express higher levels of MHC-I with respect to tumors originating from cell lines with more mesenchymal characteristics [90]. Alkalay et al. demonstrated that the acquisition of the EMT phenotype in MCF-7 human breast cancer derivatives was characterized by morphologic changes and cytoskeleton remodeling associated with an inhibition of CTL-mediated tumor cell lysis and an attenuation in the formation of the immunologic synapse between resistant cells and CTLs [91]. Furthermore, Chen et al. found that EMT was associated with escape from T cell-mediated lysis in breast cancer [38]. Targeting EMT could thus open up new opportunities for improving current immunotherapy approaches in lung cancer. We hypothesize that a combination of different immune checkpoint inhibitors (e.g., anti-PD-1/PD-L1, anti-CTLA-4, and anti-LAG-3) may be capable of impairing the mechanisms that link EMT and immune suppression.  Figure 2 provides a graphical representation of how these inhibitors target the interplay between immune cells and cancer cells with a mesenchymal phenotype. The results from ongoing studies in this area will help to confirm this hypothesis.

## 7. Conclusions

Epithelial-mesenchymal plasticity can be referred to as the different cellular states when cells are undergoing EMT and its reverse program MET and intermediate states between these two, partial EMT or hybrid EMT [92]. EMT exists in simultaneous intermediate phenotypic states, and considering the multitude of genes involved in such processes, it is important to quantitatively and qualitatively evaluate each marker, to get a full picture of the dynamic process [93].

EMT represents a crucial process in each step of the natural history of lung cancer. It is also currently under investigation as a driver of resistance to targeted therapy. Similarly, mesenchymal phenotypic changes in lung cancer cells have been acknowledged as potential mechanisms of resistance to immune checkpoint inhibitors. The literature findings presented in our review clearly indicate an intriguing link between EMT and immunotherapeutic treatment since the dynamic interplay between the TME and phenotypic changes of cancerous cells. We focused on the regulation of immune cells involved in TME and the changes in immune checkpoint expression to explore the biological mechanisms that could be exploited to develop new therapeutic strategies. Given that very little is known about the influence of this cellular mechanism on immune checkpoint inhibitor efficacy, further research is urgently needed to clarify this evolving scenario.
